# The rise and fall of rabies in Japan: A quantitative history of rabies epidemics in Osaka Prefecture, 1914–1933

**DOI:** 10.1371/journal.pntd.0005435

**Published:** 2017-03-23

**Authors:** Aiko Kurosawa, Kageaki Tojinbara, Hazumu Kadowaki, Katie Hampson, Akio Yamada, Kohei Makita

**Affiliations:** 1 Veterinary Epidemiology Unit, Division of Health and Environmental Sciences, Department of Veterinary Medicine, School of Veterinary Medicine, Rakuno Gakuen University, 582 Bunkyodai Midorimachi, Ebetsu, Japan; 2 Tojinbara Veterinary Service, 324–4 Fukutawara, Tougane, Japan; 3 Institute of Biodiversity, Animal Health and Comparative Medicine, College of Medical, Veterinary & Life Sciences, Graham Kerr Building, University of Glasgow, Glasgow G12 8QQ, United Kingdom; 4 Laboratory of Veterinary Public Health, Graduate School of Agriculture and Life Sciences, University of Tokyo, 1-1-1 Yayoi, Tokyo, Japan; Wistar Institute, UNITED STATES

## Abstract

Japan has been free from rabies since the 1950s. However, during the early 1900s several large-scale epidemics spread throughout the country. Here we investigate the dynamics of these epidemics between 1914 and 1933 in Osaka Prefecture, using archival data including newspapers. The association between dog rabies cases and human population density was investigated using Mixed-effects models and epidemiological parameters such as the basic reproduction number (*R*_0_), the incubation and infectious period and the serial interval were estimated. A total of 4,632 animal rabies cases were reported, mainly in dogs (99.0%, 4,584 cases) during two epidemics from 1914 to 1921, and 1922 to 1933 respectively. The second epidemic was larger (3,705 cases) than the first (879 cases), but had a lower *R*_0_ (1.50 versus 2.42). The first epidemic was controlled through capture of stray dogs and tethering of pet dogs. Dog mass vaccination began in 1923, with campaigns to capture stray dogs. Rabies in Osaka Prefecture was finally eliminated in 1933. A total of 3,805 rabid dog-bite injuries, and 75 human deaths were reported. The relatively low incidence of human rabies, high ratio of post-exposure vaccines (PEP) and bite injuries by rabid dogs (minimum 6.2 to maximum 73.6, between 1924 and 1928), and a decline in the proportion of bite victims that developed hydrophobia over time (slope = -0.29, se = 3, *p* < 0.001), indicated that increased awareness and use of PEP might have prevented disease. Although significantly more dog rabies cases were detected at higher human population densities (slope = 0.66, se = 0.03, *p* < 0.01), there were fewer dog rabies cases detected per capita (slope = -0.34, se = 0.03, *p* < 0.01). We suggest that the combination of mass vaccination and restriction of dog movement enabled by strong legislation was key to eliminate rabies. Moreover, the prominent role of the media in both reporting rabies cases and efforts to control the disease likely contributed to promoting the successful participation required to achieve rabies elimination.

## Author summary

Rabies is a lethal zoonosis mostly transmitted through bites by dogs infected with rabies virus. Japan has been free from rabies since the 1950s. However, there were several large-scale epidemics before its elimination. These past epidemics occurred both before and after dog rabies vaccine became available. Moreover, Japanese dog owners used to allow free roaming of dogs at that time. Therefore, studying how Japan controlled the disease will help discussion on current rabies control in endemic countries. In this study, we investigated archival data of Osaka Prefecture including newspapers between 1914 and 1933. There were two epidemics from 1914 to 1921, and 1922 to 1933. During the first epidemic, dog vaccine was not available, and the epidemic was controlled through capture of stray dogs and tethering of pet dogs. Dog mass vaccination began during the second epidemic in 1923. After these vaccination campaigns and capturing of stray dogs were conducted, rabies in Osaka Prefecture was finally eliminated in 1933. The probability of human dying from rabies declined over time, due to increased use of post-exposure vaccines. We suggest that the combination of mass vaccination and restriction of dog movement enabled by strong legislation was key to eliminate rabies.

## Introduction

Rabies has been one of the most feared diseases throughout human history and has the highest known case-fatality rate [1]. This zoonosis is mainly maintained in domestic dog populations, and continues to cause approximately 59,000 human deaths every year globally [2]. More than 99% of these deaths occur in low- and middle-income countries, where rabies is endemic in domestic dog populations [3]. However, rabies can be controlled and even eliminated from domestic dog populations using existing tools, as seen from mass vaccination programmes in Western Europe and North America [4].

**Fig 1:**
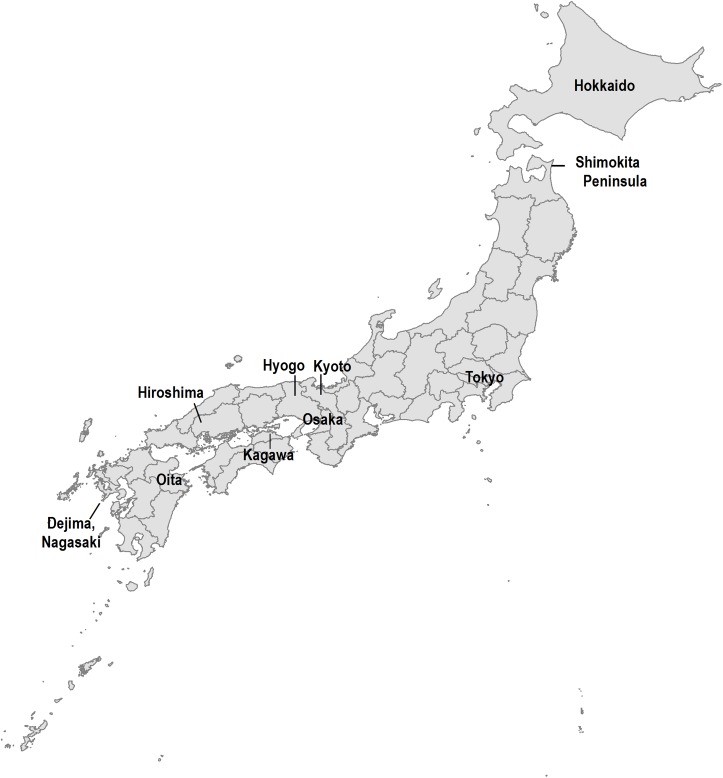
Map of Japan.

Japan was one of the first countries to eliminate rabies from domestic dog populations, and has maintained freedom from the disease ever since [5]. The history of rabies in Japan had not been well documented. Until recently, only a few articles were published in English or Japanese. The first case of dog rabies in Japan was reported in 1732 in Nagasaki, which includes Dejima, the only port allowing access from abroad during the Edo period (the era governed by Tokugawa Shogun) from 1639 to 1854 (Fig 1). This dog rabies outbreak caused many human deaths. Dog rabies rapidly spread to Hiroshima in that year, and arrived in Edo (now Tokyo) in 1736. It took 29 years for rabies to reach the north end of Honshu Island, Shimokita Peninsula in 1761, and by that time, the disease had spread to wide areas of the country [6].

**Fig 2:**
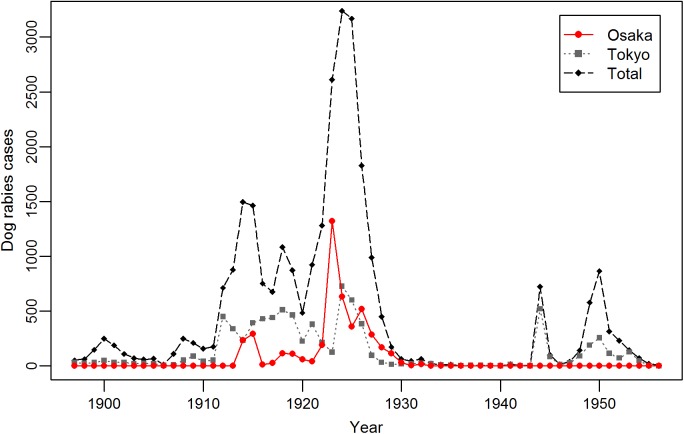
Temporal dynamics of dog rabies cases in Osaka and Tokyo Prefectures, and total numbers in Japan between 1897 and 1956, the year of elimination.

Japan opened her ports at the beginning of the Meiji era (from 1868 to 1912). Although the governance systems were modernized, there was no reporting system for rabies at the beginning of this era. In 1873, a long lasting epidemic started in Tokyo, which continued until its elimination. Rabies spread again to several parts of Japan, and in 1892 and 1893, epidemics were recorded in Oita and Nagasaki in Kyushu Island [7]. Post-exposure prophylaxis (PEP) was first administered in 1895 in Nagasaki, using attenuated vaccine produced in rabbits, in which the brain tissue of a rabid dog had been inoculated, and was successful for all the 25 treated individuals [7]. The animal infectious disease prevention law took effect in 1897, and dog rabies cases were recorded in prefectural reports since then [8]. Aggregated cases at the national level were also available since 1897 [9–10]. Although annual cases had decreased by 1906, a major epidemic that spread nation-wide began in 1907 (Fig 2). In this year, rabies entered Hokkaido Island for the first time (Fig 1).

The world’s first rabies vaccine for dogs was developed using rabbit nerve tissue in 1915, and the quantity of vaccine production and its safety were improved in 1920 by using dog nerve tissue vaccine [11–12]. The most dog rabies cases (3,205) and human deaths due to rabies (235) in Japan were recorded in 1924 [10]. Shortly thereafter, vaccine began to be used for mass vaccination, and in total 254,067 dogs were vaccinated in 1925 [13]. Dog vaccination was maintained at a similar scale over the following years; 210,515, 227,607, and 243,582 dogs were vaccinated in 1926, 1927, and 1928, respectively. This was accompanied by large-scale capture of stray dogs with 156,844, 297,396, 200,129, and 201,959 captured in 1925, 1926, 1927, and 1928, respectively [13]. Japanese people were not accustomed to chaining and leashing of dogs, or the use of muzzles before the regulation [14]. However, as seen in the Osaka Prefecture Rule for regulation of pet dogs, registration and attachment of collars became the responsibility of dog owners, and prefectures could order chaining of dogs during rabies epidemics [15], and thus most of the captured dogs were unowned. Dog rabies cases were still high with 1,799 recorded in 1926, but continued to decline thereafter [10] (Fig 2). Although dog rabies was covered in the animal infectious disease prevention law, the responsibility of dog rabies control was transferred from the Ministry of Agriculture and Commerce to the Ministry of Home Affairs under the cabinet decision in 1928 [16], because of its public health importance. Dog cases declined further, and there were fewer than ten cases annually from 1936 to 1940.

A single rabies case was detected in 1943 and in the chaos of World War II, a smaller epidemic followed (Fig 2). After the war ended in 1945, public records regarding rabies control including vaccination become available from 1949 [17]. In 1950, when numbers of dog rabies cases peaked (867), the rabies prevention law was established [10]. By law, full-time veterinary officers were placed in health centers, and authorized to pursue their responsibilities to control rabies. Before this, the police were in charge of rabies control in most prefectures, since the establishment of the animal infectious disease prevention law in 1897 [18–19]. Annual registration of dogs over 91 days old, and vaccination twice a year became the duty of dog owners, including payment of a registration fee, tax, and vaccination fee. Catching stray dogs, previously done by private companies, was placed under the direct supervision of the government, and dog catchers were given more authority to impound dogs. Free roaming dogs, even though registered, were caught in the same way as unregistered stray dogs. Use of a phenol inactivated vaccine, which was more effective than dog nerve tissue vaccine, also began in 1952. These coordinated activities were successful, and canine rabies was eliminated in 1956 [17]. The final animal rabies case detected was a rabid cat in 1957 [20]. Although rabies was eliminated in that year, vaccination of dogs is still mandatory in Japan.

There are many lessons that can be learned from the success of countries such as Japan, where rabies has been eliminated. The purpose of this study was to understand the epidemiology of rabies in Osaka Prefecture during the nationwide epidemics in the first half of the 20^th^ century using artifacts compiled from prefectural libraries and the media. Osaka Prefecture had the second largest population (2,593,701) after Tokyo Prefecture, with 2 cities, 31 towns and 263 villages in 1920 [21]. These historical records provide an unusual window into the spatiotemporal dynamics of rabies, and offer valuable lessons of relevance to the elimination of rabies from endemic countries today.

## Materials and methods

### Study site and time

Large-scale epidemics of rabies were recorded in Osaka Prefecture between 1914 and 1933, and this study investigates dog and human rabies cases in this period.

### Data collection

A database which included the annual dog rabies cases in all the 47 prefectures in Japan between 1897 and 1956, was constructed. Prefectural reports between 1914 and 1933 from libraries of Osaka and surrounding prefectures (Hyogo, Kyoto, Nara, Shiga, and Wakayama Prefectures) were investigated for animal rabies cases that occurred in Osaka Prefecture. According to a detailed description in the newspaper in 1914, although the animal species and exact method used were not indicated, confirmatory diagnosis of suspected dogs that died or were killed was carried out using experimental animals. In Kyoto Prefecture, inoculation of brain and spinal nerve tissue emulsion of a rabid dog to the brain of rabbits was a confirmatory diagnostics test [22], and Osaka Prefecture most likely used this method. For animal rabies cases, the species, date of disease onset and location found, and date and cause of death (died or killed) were recorded in these prefectural reports. Microfilms of two newspapers (Osaka-Jiji-Shinpou and Osaka-Asahi) were used to cross-validate these reports, and to supplement missing information. Annual dog rabies cases of Japan and Tokyo Prefecture between 1897 and 1937 were collected from Metropolitan Police reports [9], for comparison with the epidemic in Osaka Prefecture. Annual dog rabies cases in Japan between 1938 and 1956 were collected from a published document [10], and those of Tokyo Prefecture were collected from the public reports of Tokyo and peripheral prefectures. The purpose of the data from Tokyo Prefecture was the presentation of temporal patterns of dog rabies from the start of reporting until its elimination. The data from Japan were collected to describe the dog rabies situation in a nationwide context.

The annual number of human rabies cases (hydrophobia) in Osaka Prefecture in 1920, and between 1924 and 1928 were extracted from 1920 population census data which included infectious disease information [21], and Tokyo Metropolitan Police reports, respectively. About 50–80% of rabies patients develop hydrophobia, which is a characteristic and the most specific manifestation of the disease [23]. Rabies patients who develop hydrophobia may initially experience pain in the throat or difficulty swallowing, and particularly later in the course, hydrophobic spasms of the inspiratory muscles occur, associated with terror in attempting to swallow water [23]. In the archival reports in Japan, only ‘hydrophobia’ was recorded, and the physicians that diagnosed them, under the infectious disease prevention law, reported cases to prefectures. At that time, Tokyo Metropolitan Police received all reports of human rabies cases from throughout Japan. For the other years (1914 to 1919, 1921 to 1923, and 1929 to 1933), human cases recorded in newspapers were collected and the age and sex of victims, and the dates of exposure, symptom onset, and death were extracted.

Administrative unit shapefiles at village (*son*) level in 1919 were obtained from the National Land Numerical Information Download Service, Geospatial Information Authority, the Ministry of Land, Information, Transport and Tourism of Japan (2015) [24]. In these shapefiles, cities and towns were divided into smaller areas equivalent to the village level, and in total, there were 720 administrative units in Osaka Prefecture. The areas of administrative units were calculated, and one- kilometer buffer zones from unit boundaries were generated (see Analysis) using ArcMap version 10.1 (ESRI, Redland, USA). Demographic data were obtained from the 1920 population census in Japan [21].

The numbers of dogs vaccinated, stray dogs captured, injuries to humans caused by bites by rabid dogs, and PEP given between 1924 and 1928 in Osaka Prefecture were collected from a report of the Veterinary Investigation Center [13]. The newspapers reported the number of dog-bite injuries in Osaka Prefecture from time to time, and the information between 1914 and 1918, and 1922 and 1923 was extracted from the reports. There was no report on dog-bite injuries in newspapers for the other years.

All archival data used were publicly available at the time of the epidemics, and no sensitive data were used.

### Analysis

For the correlation between dog rabies cases in Osaka and Tokyo Prefectures, Spearman’s correlation test was performed using the records between 1897 and 1956.

We investigated how the occurrence of dog rabies cases was affected by human population density using Mixed-effects models [25]. In the first model, the logged number of dog rabies cases per km^2^ in each administrative unit was the response variable, with logged human population per km^2^ in each administrative unit a fixed effect, and year and administrative units random effects. In the second model, the logged number of dog rabies cases per capita was the response variable, with the same fixed and random effects as the first model. No dog population records were available for these administrative units.

Several dates relating to animal rabies cases were recorded in the prefectural reports including the dates of disease onset, the bite, the diagnosis, and when the animal was killed or died. The earliest date reported was used as the date of notification for analysis in this study. For available data mainly from prefectural reports, supplemented by newspaper reports, the distributions for the periods between notification and death, due to disease or being killed, were estimated by fitting gamma distributions to these intervals. Distributions of the incubation period, and the period between symptom onset and death in humans were also estimated by fitting a gamma distribution to these data extracted from newspapers using the fitdist function in R version 3.0.2 [26].

Serial intervals (*Ts*s), the periods between the date of disease onset in an index case and the disease onset in the subsequent secondary case [27], were estimated. Records of direct transmission between dogs were not available. We therefore defined an index case as the first case observed in an administrative unit where no previous rabies cases had been recorded for the preceding six months in the focal unit and surrounding units that lie within or overlap with areas of one-kilometer distance from the boundary of the focal administrative unit. Secondary cases were defined as the second case recorded in the area defined above. We considered a six-month interval without detected cases and a 1km radius, sufficient to isolate an index case as very few incubation periods are thought to exceed this interval [28] and because most distances moved by rabid dogs are thought to be less than 1 km [29]. A gamma distribution was fitted to these serial intervals. As the number of serial intervals calculated was limited, and there was no obvious temporal trend by observation, single *Ts* distribution was fitted using data between 1914 and 1933.

*R*_0,_ the average number of secondary dog rabies cases caused by a primary case in a totally susceptible population [27], was estimated from Eq 1 [30]:

$$R_{0}=1/\sum_{t=0}^{\infty }T_{s\cdot t}\lambda^{-t}$$ (Eq 1)

where *λ* is the growth rate of the epidemic curve and *t* is time. A regression with negative binomial errors was fitted to the weekly occurrence of dog rabies cases to estimate *λ*, for the epidemics from 1914 to 1921 and from 1922 to 1933, respectively. The start and end points used for the estimation of *λ* for these time series were from the week when the first case was reported after 4 weeks without any detected cases until the week with the most cases. The 4 weeks gap was used to avoid data from the last epidemic. For a partially immunized population, an effective reproductive number *R*, defined as the number of secondary infections that arise from a typical primary case when control measures are in place, could also be calculated. However when these outbreaks started, vaccination was thought to be very rare (see discussion). *R*_0_ was therefore estimated using Eq 1, sampling 5,000 times from the values between the 90% CIs of *λ* under equal probability, and from the gamma distribution of *Ts*.

The relationship between annual rabies cases in dogs and dog-bite injuries was tested using negative binomial regression, with annual cases of dog-bite injuries as the outcome variable. Negative binomial regression was chosen because the residual deviance in a Generalized Linear Model (GLM) with Poisson errors showed overdispersion (residual deviance: degree of freedom ratio was 2.2 > 1.5 [25]). The relationship between one year lagged annual dog rabies cases and dog-bite injuries was also examined, as was the relationship between annual cases of hydrophobia and dog rabies cases. We tested for temporal trends in the probability of a bite victim developing hydrophobia using GLMs with binomial errors. The sex ratio of bite victims was compared using the chi-square test based on the one-sample proportion test with continuity correction.

All calculations were performed in R version 3.0.2 [31].

## Results

### Spatio-temporal dynamics

According to the authors’ database, in 1911, dog rabies was occurring in Tokyo Prefecture and surrounding areas, East-North region, and Kyushu Island that includes Nagasaki (Fig 1). In 1912, dog rabies entered in Hyogo Prefecture, and consequently, the epidemic in Osaka and neighboring prefectures started. The proportion of prefectures with dog rabies cases was 42.6% (20/47 prefectures) in 1914, and decreased to 29.8% (14/47) in 1921. In 1922, dog rabies spread further again, and in 1923 dog rabies was present throughout most of Japan (38/47, 80.9%).

Dog rabies cases in Osaka and Tokyo Prefectures were significantly correlated throughout the period when dog rabies cases were recorded (rho = 0.516, *p* < 0.01), and regardless of the locality of epidemics, the two cities tended to have dog rabies during the same years. Osaka was the second largest city after Tokyo at that time, but in some years, dog rabies cases in Osaka Prefecture exceeded that of Tokyo Prefecture (Fig 2).

Two dog rabies epidemics occurred in Osaka Prefecture between 1914 and 1933: the first between 1914 and 1921 (879 dog rabies cases) and the second much larger epidemic lasted from 1922 to 1933 (3,705 cases, Fig 2). In total, 4,632 cases of animal rabies were reported between 1914 and 1933. Dogs accounted for 99.0% of cases (4,584), followed by 14 cattle, 14 cats, two horses, and one pig (species for 17 rabid animals were not provided). Of 4,584 dog rabies cases, owned dogs and unowned stray dogs accounted for 52.9% (2,425) and 34.5% (1,582), respectively. Information on ownership was not available for the remaining dogs (577, 12.6%).

The very first dog rabies case documented in Osaka Prefecture, was when a rabid dog bit a victim in mid June 1914 in his residence in West Ward of Osaka City. According to the newspaper report, the rabid dog died around this time, but it is not written whether the dog died of disease or was killed. The victim began showing symptoms one month later, and died of rabies three days later on July 23^rd^, 1914. Osaka Prefecture and the newspaper announced the case on July 25^th^, and control measures started thereafter. People in Osaka Prefecture were not aware that rabies was spread from rabid dogs, otherwise the police could have acted earlier potentially mitigating this epidemic. Although this first epidemic grew rapidly, cases had sharply declined by 1916.

The second larger epidemic in Osaka Prefecture escalated in 1922, and peaked in 1923 (1338 cases), while across Japan the epidemic peaked in 1924 (3205 cases) [10], before declining to the final case in 1933. After that, Osaka Prefecture was free from rabies until 1948.

**Fig 3:**
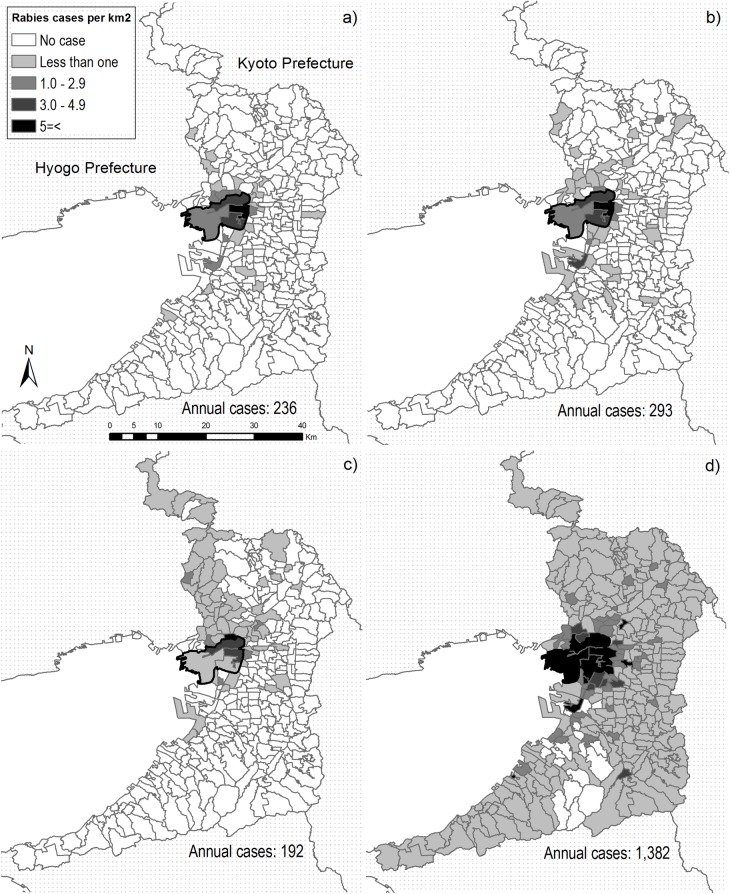
Geographical distributions of dog rabies in Osaka Prefecture in a) 1914, b) 1915, c) 1922 and d) 1923, mapped according to cases detected per km^2^.

The geographical spread of rabies between 1914 and 1915 (Fig 3A and 3B) was limited; however, during the second larger epidemic between 1922 and 1923 (Fig 3C and 3D), rabies spread across the entire prefecture. Approximately half of the dog rabies cases in the prefecture occurred in Osaka City (2,344/4,541 with the locations known; 51.6%, highlighted area with a bold line in Fig 3). In 1913, the year before the first outbreak, dog rabies cases were reported only in Hyogo Prefecture (Fig 1) among neighboring prefectures, according to the authors’ database. In 1922, dog rabies cases were reported in Hyogo and Kyoto Prefectures, and in 1923, all the neighboring prefectures had dog rabies cases.

Less than one in the legend means that there were cases with less than one case per km^2^. The boundary of Osaka City is highlighted with a bold line. The south half of left hand boundary is the coast, while the other boundaries are share borders with the other prefectures.

More cases in animals were found in areas with higher human population densities: logged dog rabies cases per km^2^ increased with logged human population density (slope = 0.66, se = 0.03, *p* < 0.01, Table 1). While the higher the (logged) human population density, the fewer (logged) animal rabies cases were detected per capita (slope = -0.34, se = 0.03, *p* < 0.01, Table 2).

**Table 1:** Mixed-effects model results for the relationship between logged dog rabies cases per km^2^ and logged human population density.

Fixed effect variables	Estimate	Standard error	p-value
Intercept	-6.14	0.25	<0.01
Human population density per km^2^	0.66	0.03	<0.01
Random effect variables	Variance	Standard deviation	
Year	0.09	0.30	
Administrative units	0.43	0.65	
Residual	0.45	0.67	

**Table 2:** Mixed-effects model results for the relationship between logged dog rabies cases per capita and logged human population density.

Fixed effect variables	Estimate	Standard error	p-value
Intercept	-6.14	0.25	<0.01
Human population density per km^2^	-0.34	0.03	<0.01
Random effect variables	Variance	Standard deviation	
Year	0.09	0.30	
Administrative units	0.43	0.65	
Residual	0.45	0.67	

**Fig 4:**
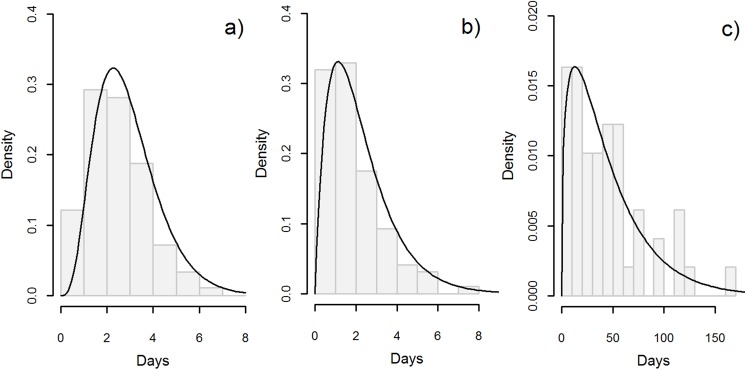
Frequency distributions for: a) the infectious period between rabies onset and death in rabid dogs in days; b) the infectious period between rabies onset and when rabid dogs were killed in days; and c) the serial interval between identified primary and secondary cases in days.

The mean infectious period for dogs that died of rabies was 3.3 days (90%CI: 0.06–11.4, Fig 4A), whereas for those that were killed it was 2.4 days (90%CI: 0.3–6.3, Fig 4B). The mean serial interval between identified primary and secondary cases was 45.0 days (90%CI: 4.7–118.8, Fig 4C).

The growth rate, *λ*, of the first epidemic was 1.03 (90%CI: 1.018–1.037), and was 1.01 (90%CI: 1.010–1.011) for the second epidemic (Table 3). Given the observed serial intervals, *R*_0_ of dog rabies was estimated as 2.42 (90%CI: 1.94–2.91) for the first epidemic, and 1.50 (90%CI: 1.48–1.52) for the second (Table 3).

**Table 3:** Epidemiological parameters estimated.

Item	Distribution	Mean (median)	90%CI	N
***Dog rabies***				
Period between disease onset and death in days	Gamma (shape = 4.56, rate = 1.57)	2.9 (2.7)	1.1–5.5	181
Period between disease onset and date when killed in days	Gamma (2.05, 0.92)	2.3 (1.9)	0.4–5.2	97
Serial interval (*Ts*) in days	Gamma (1.41, 0.03)	45.0 (34.7)	4.7–118.8	49
Growth rate (*λ*) (1914)		1.03	1.018–1.037	15 weeks
Growth rate (*λ*) (1921–22)		1.01	1.010–1.011	65 weeks
*R*_0_ (1914)		2.42 (2.42)	1.94–2.91	5000 iterations
*R*_0_ (1921–22)		1.50 (1.50)	1.48–1.52	5000 iterations
***Human rabies***				
Incubation period	Gamma (0.84, 0.01)	76.4 (49.1)	2.5–242.9	13
Infectious period	Gamma (4.31,1.62)	2.7 (2.5)	1.0–5.1	9

### Public health impacts

A total of 3,805 injuries due to bites by rabid dogs were reported between 1914 and 1933 in Osaka Prefecture. Seventy-five human rabies deaths were reported, corresponding to 2.0% (75/3,805) of bite victims. As mentioned above, the public became aware of rabies after the announcement of the first human rabies death on July 25^th^, 1914. In this first year, 13 people died of rabies.

**Fig 5:**
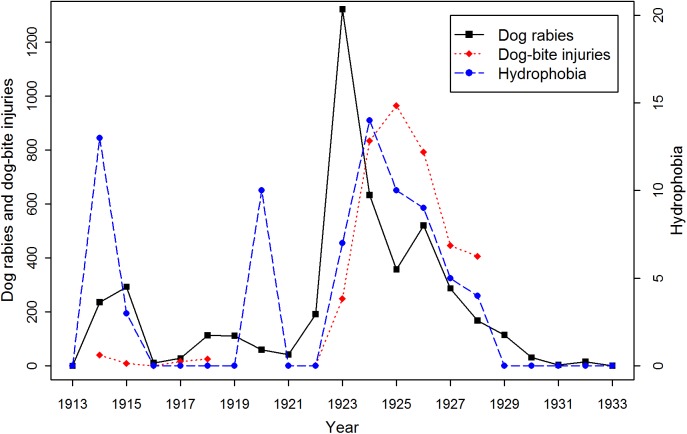
Time series of dog rabies cases, dog-bite injuries, and hydrophobia in Osaka Prefecture. The numbers of dog bite injuries were not recorded in 1913, between 1918 and 1921, and between 1929 and 1933. There were no recorded human rabies deaths in 1913, between 1916 and 1919, between 1921 and 1922, and after 1928.

**Fig 6:**
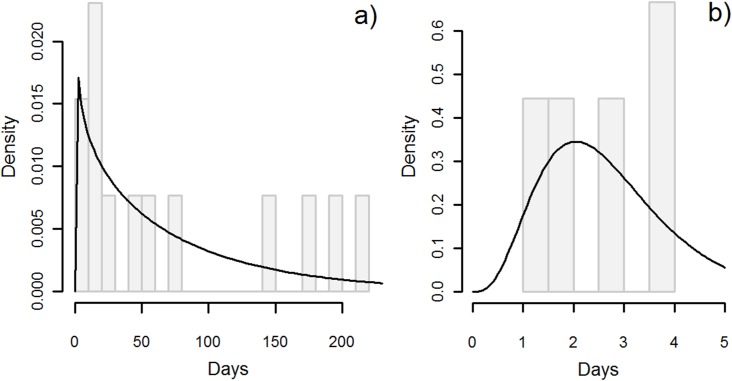
Distributions for human rabies: a) incubation period in days; and b) infectious period in days.

Time series of dog rabies cases, dog-bite injuries, and hydrophobia in Osaka Prefecture (Fig 5) showed similar temporal patterns of increase and decrease, but dog-bite injuries and hydrophobia were lagged during the second epidemic. Annual cases of dog-bite injuries had significant positive relationships with dog rabies cases that year (slope = 0.003, se = 0.001, *p* = 0.01), and the previous year (slope = 0.004, se = 0.001, *p* = 0.01). Annual cases of hydrophobia also had a significant positive relationship with dog rabies cases (slope = 0.003, se = 0.0002, *p* < 0.01). There were less human rabies deaths during the second epidemic, with no reports of hydrophobia between 1916 and 1919. The mean number of deaths during years when deaths occurred was 8.1 (range: 3–14), and the mean incidence was 0.31 deaths per 100,000 persons (range: 0.11–0.54). The most deaths, 14, were recorded in 1924. The proportion of dog-bite victims that developed hydrophobia each year significantly declined over time (slope of logit = -0.29, se = 0.03, *p* < 0.01). There were no reports of hydrophobia or the number of dog-bite injuries in newspapers between 1929 and 1933. The mean incubation period and infectious period in humans was 76.4 days (90%CI: 2.5–242.9, estimated from 13 cases, Fig 6A) and 2.7 days (90%CI: 1.0–5.1, estimated from nine cases, Fig 6B), respectively (Table 3).

**Fig 7:**
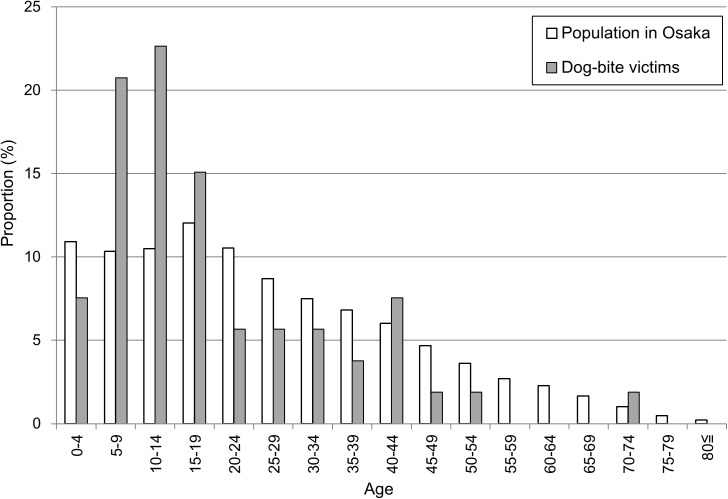
Age distributions of dog-bite victims and the general population in Japan (1914–1933).

The sex of dog bite victims was rarely recorded (73 cases, 1.9%), but of these, significantly more men (49, 67.1%) were bitten than women (24, 32.9%, *x*^2^ = 7.9, df = 1, *p* < 0.01). The age of dog-bite victims was recorded in just 49 cases (taken only from newspaper records). The median age of these bite victims was 13 (mean 19.4), with 49% in children 5–14 years old. Most bites were in the 10–14 year age group, followed by the 5–9 and 15–19 year age groups (Fig 7).

### Control measures in Osaka Prefecture

Table 4 shows rabies control measures taken in Osaka Prefecture between 1914 and 1933. On the day of the announcement of the first human death due to rabies in Osaka Prefecture, July 25^th^ 1914, police started culling stray dogs, and 150 stray dogs were culled by July 31^st^. Police organized health checks of dogs by veterinarians examining for rabies based on clinical signs, and marking checked dogs with a red color. Victims of dog-bite injuries by suspected rabid dogs were encouraged to receive PEP for free. On January 4^th^ 1915, Osaka Prefecture Rule for regulation of pet dogs was revised, and registration of dogs over three months old, attachment of dog collars with a name and address, and chaining or application of muzzles to dogs with biting tendencies became the duty of dog owners. The rule also stated that dogs must be chained when the authority orders during dog rabies epidemics, and otherwise free roaming dogs would be regarded as unowned stray dogs [15]. As rabies vaccine for dogs was not available in this epidemic, rabies control was based on keeping pet dogs chained, and on mass culling of stray dogs. According to Osaka Prefecture Notices and newspaper articles, Osaka Prefecture government announced dog chaining orders several times during the epidemics.

**Table 4:** Rabies control measures taken in Osaka Prefecture between 1914 and 1933.

Year	Activities	Source
1914	Culled 150 stray dogs from July 25th; health check of dogs by veterinarians for clinical signs and mark checked ones with red color; free PEP provided to victims of dog-bite injuries	Newspaper (Jul 31)
	Diagnosis of dog rabies by inoculation of brain tissue emulsion to rabbits; emergency order to chain owned dogs	Newspaper (Oct 20)
	Culling of stray dogs (over 30,000) with 20,000 more estimated to be present	Prefecture Notice (Nov 3), Newspaper (Dec 12)
1915	Registration of dogs; reports of births, movements, and deaths; application of a collar with name and address; application of muzzle to aggressive dogs (revision in Osaka Prefecture Rule for regulation of pet dogs)	Prefecture Order (Jan 4)
	Extension of emergency order to chain owned dogs	Newspaper (Mar 17)
	Communication to raise awareness of rabies; encouragement of reporting rabies suspected dogs	Prefecture News (May 26)
	Owners who do not keep dogs chained ordered to serve prison sentence	Newspaper (Jun 21)
	Capture and culling of any stray dogs	Newspaper (Jul 16)
	Since control began, 36,399 stray dogs captured; 14,389 dogs registered	Newspaper (Oct 22)
1916	Notice of the dates and places of mass stray dog capture activities	Prefecture Notice (Jun 7)
1919	Ban of dogs in Osaka Prefecture to enter Shodo area in Kagawa Prefecture (notice by Kagawa Prefecture)	Prefecture Notice (Oct 30)
1922	Act on domestic animal infectious disease control established	Iwabuchi, 1970
	Ban of inter-prefectural dog movement from rabies affected prefectures for dogs not vaccinated in the last six months	Prefecture Notices in 1922 and 1923
1923	Registration, chaining dogs, and leashing dogs while walking are owners’ responsibility; any stray dogs to be killed	Prefecture Notice (Apr 19)
	Mass vaccination starts in June	Prefecture Notice (May 21)
1925	Notices of mass vaccination	Peripheral prefectures of Osaka
	Reward for capturing stray dogs	Newspaper (May 27)
1926	Recognition of four veterinarians for rabies control Announcement of rabies vaccination week; reward for capturing stray dogs	Prefecture News (Feb 11) Newspaper (Sep 12)
1928	Notice of mass vaccination	Prefecture Notice (Jan 16, Feb 27, Apr 2, and May 24)
1929–1933	Repeated notices of dog rabies mass vaccination	Prefectural Notices

In 1919, when dog rabies cases began increasing, a ban of dog movement from prefectures with rabies cases was applied. In 1922, the Act on domestic animal infectious disease control (a current act with the same name was established in 1951) was established, and dog vaccination became compulsory for inter-prefectural dog movement. Mass vaccination started in 1923, and a certificate of vaccination effective for one year was provided to owners. In 1923, the order of chaining dogs, and leashing for walking was announced again. If not, dogs were regarded as strays, and were killed. In 1925 and 1926, a reward for capturing stray dogs was announced. Until 1933, a combination of dog vaccination and restriction of free movement of dogs continued.

Table 5 shows the numbers of dogs vaccinated, captured stray dogs, dog-bite injuries by rabid dogs to humans, and PEP administered in Osaka Prefecture between 1924 and 1928. The number of PEP and the rate of PEP delivered to dog-bite injuries was high, particularly in 1928. No records of PEP use were available for other years. If the ratio of human deaths to dog rabies cases in 1914 (13 deaths: 236 dog rabies cases = 1: 18.2) had continued, the epidemic between 1914 and 1933 could have caused around 252 deaths (252: 4,584 dog rabies cases), compared with 75 recorded deaths.

**Table 5:** The numbers of dogs vaccinated, stray dogs captured, dog-bite injuries by rabid dogs, and PEP in Osaka Prefecture between 1924 and 1928.

Year	Dog vaccination	Capture of stray dogs	Dog-bite injuries by rabid dogs	Post-exposure prophylaxis	Ratio of PEP and dog-bite
1924	Not provided	Not provided	835	6,683	8.0
1925	26,386	1,224	965	6,014	6.2
1926	13,893	31,720	793	7,480	9.4
1927	15,634	13,229	447	5,804	13.0
1928	23,431	21,987	406	29,869	73.6

## Discussion

Here we describe the epidemiology of rabies in Osaka Prefecture, during a period when nationwide epidemics were occurring in Japan. For the analyses, the most reliable data were collected, as far as possible. However, there is a limitation in data accuracy. The biggest challenge of data collection was the lack of human rabies records in the years without special attention to rabies. By law, human cases with hydrophobia were reported to Osaka Prefecture, but unfortunately, prefectural annual hygiene reports during the period were lost, according to our archive survey. The records were not stored in the Ministry of Health, Labour and Welfare as well. Moreover, as mentioned in the materials and methods, hydrophobia is seen in 50–80% of rabies patients, and other types of illness such as paralysis (about 20% of the patients) [23] may not have been reported. Also, a report of hydrophobia was based on the notification by a physician, and it may not have involved biological confirmation. Nevertheless, newspapers not only reported each single case, but also presented summary reports from time to time, which contributed greatly in filling data gaps. On the other hand, although there might be underreporting, dog rabies cases were confirmed by the inoculation of brain tissue emulsion to rabbits [22].

During the times of special attention to rabies, dog rabies reports to Osaka and neighboring prefectures were thought to be accurate even in rural areas, considering strong enforcement of dog registration and mandatory reporting of suspected rabies cases accompanied by penalties in case of failure, and information sharing between neighboring prefectures. However, dog rabies cases without clear furious symptoms may not have been reported, and unowned rabid dogs may have been died in remote areas unnoticed. This potential underreporting may have caused underestimation of *R*_0_.

Of the two major epidemics in Osaka Prefecture, the second was more severe than the first, in terms of both geographic spread and numbers of dog rabies cases. However, *R*_0_ was significantly larger in the first epidemic (2.42) than the second (1.50, Table 1) and larger than previously recorded elsewhere in the world [29]. Our estimate of *R*_0_ is potentially conservative, since we used the serial interval from Osaka, which was longer than that reported from Tanzania [29] (45.0, 90%CI: 4.7–118.8 vs 24.9, 95%CI: 23.7–26.2, and our method of estimating the serial interval may have been biased towards longer intervals because direct transmission was not observed). The first case of dog rabies in Osaka Prefecture in 1914 occurred in the West Ward of Osaka City, which shares a boundary with Hyogo Prefecture, and dog rabies was considered to be introduced from this neighboring prefecture. Since this was the first large-scale rabies epidemic in the history of Osaka Prefecture, lack of awareness about rabies may have resulted in such a large number of cases. The high growth rate in the first epidemic in 1914 might be also due to fewer case reports during the initial phase because of a lack of awareness. There was a one-month period between the dog-bite injury and the onset of hydrophobia in the first victim in Osaka Prefecture. During this time considerable undetected transmission likely occurred leading to rapid spread in this entirely susceptible population. Furthermore, as shown in Table 4, an estimated population of 50,000 unconstrained stray dogs at that time likely exacerbated spread.

As shown in Fig 4B, sometimes it was difficult to kill free roaming rabid dogs. The longest time between illness onset until a rabid dog was killed was 8 days. However, the mean infectious period of dogs that were killed was short (2.4 days). This rapid response may explain the long serial interval and moderate *R*_0_ calculated. Moreover, once rabies control started, stray dogs were rapidly captured, and most of these were captured in 1914. Dog owners seemed not to have had a habit of keeping dogs chained before the epidemic, because Osaka Prefecture repeatedly communicated this message to the public through prefecture notices and media. Strict enforcement with a punishment, in case pet dogs were not kept chained, in 1915 may have been effective. The number of dog rabies cases sharply declined in 1916, which suggests that human responses to encounters with rabid dogs may have reduced epidemic growth, as has been previously noted [32].

A ban of inter-prefectural dog movement in limited areas in 1919 seemed ineffective for rabies control, and the nation-wide epidemic grew from 1921. The second epidemic was widespread and appeared to involve neighboring prefectures. In 1922, the animal infectious disease prevention law was established [10], and dog vaccination became available for inter-prefectural movement of dogs. However, this still did not slow the epidemic, probably because it was already widespread, there were large numbers of stray dogs with high rates of turn-over, and low compliance with registration and tethering dogs. A newspaper on 1922 June 29th estimated that there were 10,000 unregistered and 20,000 stray dogs in Osaka Prefecture. Comparing descriptions in newspapers between 1914 and 1916, with those between 1919 and 1922, mass culling of stray dogs appeared more frequently in the former years, but not in the latter. This may have reflected the expectation towards efficacy of vaccine for dogs. Mass vaccination in Osaka Prefecture started in 1923, at the peak of the epidemic, and annual dog rabies cases declined sharply as a result. However, dog rabies cases increased again in 1926. With the restart of capturing stray dogs, rabies was finally eliminated from Osaka in 1933, suggesting mass vaccination alone as applied during this epidemic may not have been sufficient to eliminate dog rabies. Information on the dog population in 1920s’ is unfortunately not available, and vaccination coverage may not have exceeded the immunity threshold.

The number of dog rabies cases per unit area was positively correlated with human population density. However, more information on whether transmission rates (and *R*_0_) scale with dog population density is needed to determine whether transmission is density or frequency-dependent. Unfortunately, no data were available on the size of the dog population at administrative levels. The negative relationship between human population density and dog rabies cases per capita found in this study implies that the dog: human ratio may be lower in densely populated areas i.e. more dog ownership or more dogs per human in less densely populated (or more rural) areas.

Although large numbers of dog rabies cases were reported, the incidence of human rabies deaths remained low. The range of annual human rabies incidence (0.11 to 0.54 deaths per 100,000) was far lower than in Tanzania (4.9 deaths per 100,000) estimated using active surveillance [33], but is comparable with the situation in Bali in 2009. Between December 2008 and November 2009, the incidence of human rabies deaths was 0.51 per 100,000 persons (20/3,890,757) in Bali, Indonesia, and in the following 12 months, incidence increased to 2.13 deaths per 100,000 (83/3,890,757) [34–35]. Moreover, the probability of developing hydrophobia after a dog bite decreased over time. The PEP used at that time already seemed to have high efficacy: 10–15% of unvaccinated patients bitten by rabid dogs died, whereas only 0.25–1% of patients vaccinated with the Pasteur dry toxin vaccine method died in Tokyo Prefecture [36]. As shown in Table 5, large numbers of PEP were administered to dog bite victims, and the number increased during the last part of the epidemic. The big differences between the number of dog-bite injuries by rabid dogs and PEP suggested that the victims of dog-bite injuries may have sought PEP immediately regardless of the status of infection of the dog. In the record of discussion at the transition of responsibility of rabies control from the Ministry of Agriculture to the Ministry of Home Affairs in 1929, it is written that human rabies was almost eliminated from Japan in 1928 [37]. The increased PEP use in 1928 (Table 5) may reflect this transition of responsibility of rabies control, though a year earlier. Based on the ratio of hydrophobia cases and dog rabies cases in 1914, if PEP use had remained at initial levels, 252 humans rabies deaths would have occurred during the two epidemics in Osaka Prefecture. However, these data suggest that over 170 lives may have been saved by the increased PEP use. The time lag between the incidence of dog rabies cases and dog bite injuries might also reflect increased awareness of rabies over time, and consequently, improved reporting of dog bites. In fact, reported dog-bite injuries could include those by healthy as well as by rabid dogs.

Although there is limited information on dog bite victims, more men were bitten than women and children were more at risk than adults. Previous studies have also revealed that being male or a child is a risk factor for bite injuries from rabid dogs and rabies infection [3, 34, 38].

In conclusion, this paper provides a useful example of the elimination of canine rabies. Authorities raised awareness of rabies and encouraged victims of dog bite injuries to receive PEP, saving many lives. Mass vaccination of dogs undoubtedly played a critical role in controlling and ultimately eliminating rabies. Authorities also aimed to reduce the number of stray dogs using a reward scheme, and enforced the tethering of pet dogs, even while walking, which was not common in Japan at that time. Strict legislation was important in dramatically changing the behavior of dog owners.

A newspaper described the first case on July 26, 1914 vividly; ‘*his wife dropped a coin on the floor and it slipped off through mats and floor woods*. *When he raised up the mat and was trying to pulling the coin with a bamboo stick*, *suddenly a dog rushed from the floor space*, *and bit his left finger strongly*. *The stray dog hid in the floor space during the day time*, *and strayed and bit anything in front of him during nights*.’ And the article ends with this; ‘*we strongly request police to make efforts in killing stray dogs*, *and advise people to assume any dogs to be rabid*’.

Such views on strict stray dog population control may not be accepted, in terms of culture and animal welfare, in current rabies endemic countries. However, the effective control options recommended nowadays–vaccination and improvements in dog husbandry—have not been changed since almost a century ago, especially after dog vaccine came in to use. Although socioeconomic conditions may be challenging in some rabies endemic countries and regions, and the findings in past Osaka Prefecture may not be directly comparable with these places, the elimination of rabies is surely possible, by well-coordinated implementation of control programs with participation of all stakeholders from local communities to global partners. Finally, our experience of rabies in Japan suggests that the media can play an important role in reporting the dangers of rabies and promoting participation in rabies control activities to ensure progress towards elimination.
